# Immunohistochemical Expression of Nanog and Its Relation with Clinicopathologic Characteristics in Breast Ductal Carcinoma

**DOI:** 10.29252/.23.3.184

**Published:** 2019-05

**Authors:** Omid Emadian Saravi, Farshad Naghshvar, Zhila Torabizadeh, Somayeh Sheidaei

**Affiliations:** 1 Department of Pathology, Faculty of Medicine, Mazandaran University of Medical Science, Sari, Iran; 2 Department of Pathology, Gastrointestinal Cancer Research Center, Mazandaran University of Medical Science, Sari, Iran

**Keywords:** Breast cancer, Immunohistochemistry, Nanog homeobox protein

## Abstract

**Background:**

Cancer stem cells (CSCs) are a group of tumor cells with self-renewal property and differentiation potential. CSCs play a crucial role in malignant progression of several types of tumors. However, what is still controversial is the clinicopathological relationship between the Nanog marker and its prognostic value in the patients with breast cancer. The expression of Nanog in the patients with breast cancer and its correlation with clinicopathological prognostic factors was explored in the present study.

**Methods:**

A sample of 120 breast cancer tissues was obtained from the patients who referred to Imam Khomeini Hospital in Sari City, Iran during January 2012 and December 2016. The associations between Nanog expression and clinicopathological factors were analyzed based on immunohistochemical analysis.

**Results:**

The expression of Nanog was detected in 67 (55.8%) patients with a high expression rate in 24 (36%) cases (staining index ≥3). Moreover, there was a statistically significant relationship between Nanog expression and clinicopathological factors, including tumor grade (*p* = 0.001), lymph node metastasis (*p* = 0.01), and the stage of the disease (*p* = 0.003).

**Conclusion:**

Findings of the study indicate that Nanog may act as a biomarker for prognostic prediction in patients with breast cancer.

## INTRODUCTION

Breast carcinoma is the most common malignant tumor with the highest mortality rate in women. It involves more than 1.7 million cases around the world annually[1]. Despite improvements in cancer treatment, low overall survival rate is still found in patients with breast cancer. However, recurrence and distant metastasis after surgical resection of primary tumor are often incurable and fatal, leading to poor prognosis of breast cancer[2]. Cancer stem cells (CSCs) are potential players in the pathogenesis and development of malignant tumors, and this issue has been investigated by researchers in recent years[3,4]. CSCs are small populations of neoplastic cells within the tumor bulk with self-renew ability, which can create new tumors[5]. Currently, there is a consensus on the role of CSCs in progression, metastasis, and recurrence of various types of tumors[6,7]. Embryonic stem cells (ESC) have shown the same characteristics as the CSCs, which may indicate a similar mechanism in cancer development[8,9]. Dysregulated proliferation can be the underlying molecular mechanism of early embryo self-renewal reactivation[10].

Nanog is a key multidomain homeobox transcription factor for maintaining ESC pluripotency[11,12]. Human Nanog gene is located on the chromosome region 12p13.31 and codes for a 305 amino-acid protein with conserved homeo-domain motif localized to the nucleus[13]. It has been known that Nanog acts in maintaining the undifferentiated state of pluripotent stem cells. The differentiation-promoting signals induced by the extrinsic factors, leukemia inhibitory factor/bone morphogenetic protein, leukemia inhibitory factor, and Stat3 are counteracted by Nanog expression[14]. Nanog is a key protein that binds Rex-1 promoter and regulates the expression of this pluripotent marker. Nanog knockdown in ESCs leads to lower Rex-1 expression; however, forced expression of the protein induces Rex-1 expression[15]. The cell differentiation can be promoted when Nanog expression is down-regulated. Transcription factors Oct4, SOX2, FoxD3, and Tcf3 and tumor suppressor p53 contribute to the regulation of Nanog expression[16]. Nanog is expressed in various types of malignancies, including brain tumors, breast cancer, and colorectal carcinoma[14]. The present study explored Nanog expression in patients with breast cancer and also its relationship with clinicopathological prognostic factors.

## MATERIALS AND METHODS

### Sampling

The specimens of breast cancers were obtained from 120 patients referred to Imam Khomeini Hospital in Sari (Iran) during January 2012 and December 2016. Clinicopathologic parameters were age, tumor size, histological grade, perineural invasion, vascular invasion, lymph node metastasis, and tumor stage. Data were gathered using hematoxylin and eosin (H&E)-stained pathologic slides, pathological records, and hospital files. All the patients were women, with the mean age of 54.5 (ranging from 28 to 77) years. The samples were taken from the cancerous and adjacent normal tissues. For microscopic examination, the tissues were routinely fixed with formalin 10% before being embedded in paraffin.

### Ethical statement

This research was performed using the samples stored after the pathological diagnosis. All the data were obtained from anonymous samples. Mazandaran University of Medical Science (Mazandaran, Iran) approved the study (ethical code: IR.mazums.rec. 95/1863).

### Inclusion and exclusion criteria

Participants of the study included patients diagnosed with invasive ductal carcinoma following the breast surgery and those who did not receive neoadjuvant treatment. The inappropriate paraffin tissue blocks for immunohistochemical staining as well as those samples with incomplete documents were excluded from the study.

### Immunohistochemistry (IHC) procedure

Tissue sections with 4 micrometer thickness were prepared and stained by H & E for histological evaluation, and representative blocks were chosen for immunohistochemical study. Absolute ethanol and 96% ethanol were used in three steps in order to eliminate paraffin-xylene solution. The slides were rinsed under running water, dried and transferred to 1% hydrogen peroxide mixture. Methanol was added to the target solution after 10 min. In order to reach the boiling point, the slides were first transferred to autoclave with 1*00* °*C* for 13 min and then removed and put aside to reach room temperature. The tissue was washed with both running water and wash buffer. Next, the slides were incubated at envision for 60 minutes using diagnostic kit for monoclonal Nanog with 1/500 dilution, and then they were washed twice with wash buffer. The DAB solution was added and after appearing brown color, the slides were placed again in wash buffer for two minutes. Finally, the washed slides were stained with Mayer's hematoxylin, rinsed in distilled water, fixed in xylol and mounted with Entellan. Positive control kit for Nanog was Seminoma tissue. Our negative control was the tissue that the primary antibody did not shed. The nuclear staining was observed and scored by two pathologists according to the published criteria using a semi-quantitative score[17].

### Scoring

**Fig. 1:**
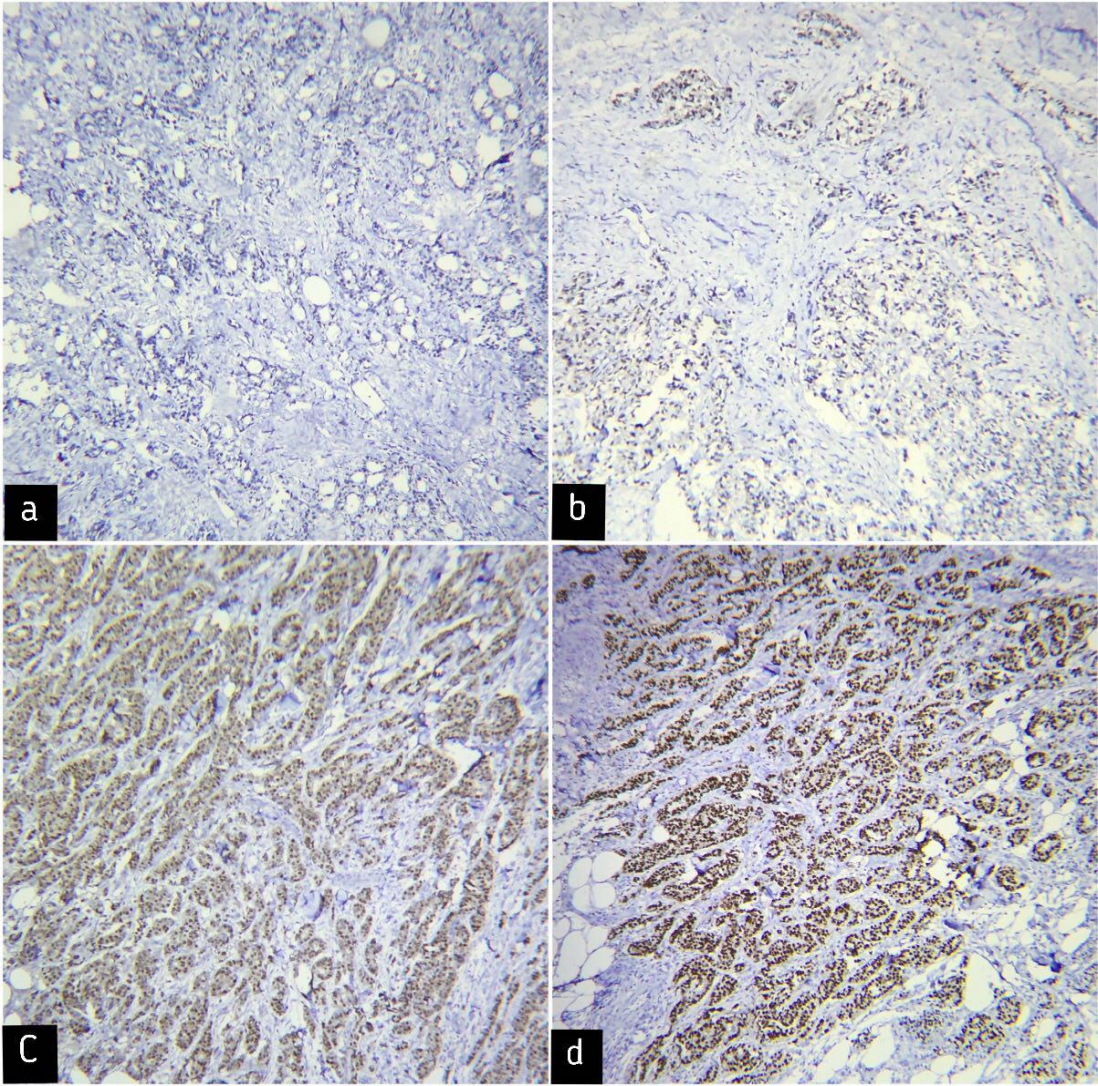
Immunohistochemical staining of Nanog showing different expression levels in breast cancer samples (magnification 100×). (a) Negative staining for Nanog (score 0), (b) weak staining for Nanog (score 1), (c) moderate staining for Nanog (score 2), and (d) severe staining for Nanog (score 3).

For the evaluation of IHC results, the tumor cell staining intensity was measured using four scores as 0 (no staining), 1 (weak staining), 2 (moderate staining), and 3 (strong staining), as indicated in Figures 1a, 1b, 1c, and 1d, respectively. Distribution of expression was also scored as 0 (none of tumor cells), 1 (1-50% of positive tumor cells), and 2 (50-100% of positive tumor cells). The total score was calculated by multiplying the percentage of cell staining by the staining intensity. Tumors with low and high expression had total scores of 0-2 and 3-5, respectively.

### Statistical analysis

SPSS 22 was employed to analyze the data. Fisher’s exact and chi-squared (X^2^) tests were used to analyze the significance of the relationship between clinicopathological characteristics and Nanog expression. A *p* value of less than 0.05 was considered as statistically significant.

## RESULTS

Samples of this study were 120 cases of invasive breast carcinomas. The mean age of the breast cancer patients was 54.5 at the time of their cancer diagnosis. The study detected Nanog by IHC staining in 67 samples of breast carcinomas (55.8%). With regard to the degree of tumor differentiation, most of the cases were grade II (68 [56.7%]), followed by 33 (27.5%) as grade I and 19 (15.8%) as grade III of the tumor. Sixty-seven of the breast cancer patients (55.8%) of the study exhibited lymph node involvement. The higher Nanog expression was observed in 44 lymph node positive samples (36.7%).

Table 1 briefly reports the association between clinicopathological parameters and the expression of Nanog. A significant correlation was found between Nanog expression and microscopic grade (*p* = 0.001), tumor stage (*p* = 0.003), and lymph node involvement (*p* = 0.01) in breast cancer samples. There was not any relationship between Nanog expression and age (*p* = 0.71), tumor size (*p* = 0.25), perineural invasion (*p* = 0.06), and vascular invasion (*p* = 0.27).

**Table 1:** Relationship between the expression of Nanog and clinicopathological factors

Clinicopathological factors	No. of positive tumor cells (%)	No. of negative tumor cells (%)	*p* value
Age (years)			
50>	34 (28.3)	25 (20.8)	0.717
50≤	33 (27.5)	28 (23.3)
Tumor size			
2 cm<	13 (10.8)	17 (14.2)	0.253
2-5 cm	40 (33.3)	25 (20.8)	
5 cm <	14 (11.7)	11 (9.2)
Histological grade			
1	5 (4.2)	28 (23.3)	0.001
2	51 (42.5)	17 (14.2)	
3	11 (6.7)	8 (6.7)
Perineural invasion			
positive	26 (21.7)	30 (25)	0.066
negative	41 (34.2)	23 (19.2)
Vascular invasion			
positive	35 (29.2)	22 (18.3)	0.273
negative	32 (26.7)	31 (25.8)
Nodal status			
positive	44 (36.7)	23 (19.2)	0.017
negative	23 (19.2)	30 (25)
Stage			
1	6 (5)	17 (14.2)	0.003
2	26 (21.7)	20 (16.7)	
3	35 (29.2)	16 (13.3)	

## DISCUSSION

In this study, 55.8% of tumoral sample have expressed Nanog marker. Finicelli *et al*.[18] also reported Nanog expression in 44.5% of breast cancer patients that is close to our result. However, Nagata *et al*.[17] found little (9.8%) expression and Ezeh *et al*.[19] observed no expression of Nanog in breast cancer cells. These differences in Nanog detection could result from different sensitivities of the methods used for assessing the Nanog expression. A number of studies have confirmed that Nanog is rather expressed in most patients with breast tumors compared to the individuals with normal tissues[14,19]. In addition, its expression was linked to CSC-like properties[20], tumor aggressiveness[21], hormone resistance[17], and chemotherapeutic agents[22].

We identified Nanog protein to be predominately expressed in the nucleus of tumor cells. IHC analysis of Nanog in breast carcinoma tissues has shown both nuclear and cytoplasmic localization of this protein; a result that is compatible with ours[17,19]. Our study also found a significant association between Nanog expression in tumor cells and several clinicopathologic factors. These factors included lymph node metastasis, stage of the disease, and histological grade. However, no relationship was detected between Nanog expression and age, tumor size, and neurovascular invasion. Finicelli *et al*.[18] and Wang *et al*.[23] have demonstrated a significant connection between tumor grade and Nanog expression in the majority of the patients who were diagnosed as grade II and grade III, respectively. We also obtained the same result but with patients in grade II. Similar to the findings of Ezeh *et al*.[19], we found a significant correlation between tumor, node, metastases stage 3 of the disease and the expression of Nanog marker. However, Nagata *et al*.[17] and Wang *et al*.[23] did not observed any link between the stage of the disease and Nanog expression[17,23]. With regard to the tumor size in our study, no significant correlation existed between Nanog expression and tumor size. Nevertheless, Wang *et al*.[23] have demonstrated a significant association between these two variables. It has been proposed that Nanog overexpression is related to resistance to hormone or anticancer therapy in breast cancer[20]. Arif *et al*.’s[24] results showed that Nanog plays a role in tumorigenesis and affects the resistance to tamoxifen and has an inverse relationship with the expression of estrogen receptor and the apoptosis pathway. We showed that Nanog overexpression is clearly restricted to tumor cells, thus confirming the findings of Ezeh *et al*.[19]. In line with our study, Jin *et al*.[25] and Wang *et al*.[23] have revealed a significant correlation between the high expression of Nanog in breast cancer tissues and the higher rate of lymph node metastases. However, Finicelli *et al*.[18] and Wang *et al*.[23] did not find a significant relationship between Nanog expression and patients’ age; this result was in agreement to our finding.

In this study, we found no significant link between perineural and vascular invasion. However, there is no study reporting these two prognostic factors. Nagata *et al*.[26] have shown that the overall survival was significantly low in breast cancer patients with Nanog overexpression, and thus the expression of Nanog might be a poor prognosis factor for all breast cancer subtypes. Finally, in a study by Finicelli *et al*.[18], no significant correlation was found between Nanog expression and clinical outcome.

Some limitations of this study include short follow-up period, evaluation of distant metastasis, and survival rate of the patients. This study found a strong connection between Nanog expression and some clinicopathologic features in the patients with breast cancer, which includes lymph node metastasis, stage of the disease, and grade of disease. Our findings indicate that there is an association between the expression of Nanog and prognosis of the breast cancer patients. Moreover, worse prognostic characteristics were observed in the patients with high expression of Nanog. However, controversies exist among the studies conducted to evaluate this relationship.
